# Simple Synthesis of Cellulose-Based Nanocomposites as SERS Substrates for In Situ Detection of Thiram

**DOI:** 10.3390/nano14110987

**Published:** 2024-06-06

**Authors:** Boya Shi, Lian Kan, Yuliang Zhao, Shangzhong Jin, Li Jiang

**Affiliations:** College of Optical and Electronic Technology, China Jiliang University, Hangzhou 310018, China; s21040803032@cjlu.edu.cn (B.S.); kannnliannn@163.com (L.K.); p23040854168@cjlu.edu.cn (Y.Z.)

**Keywords:** SERS, thiram, flexible substrates

## Abstract

There is a growing interest in the use of flexible substrates for label-free and in situ Surface-enhanced Raman Spectroscopy (SERS) applications. In this study, a flexible SERS substrate was prepared using self-assembled Au/Ti_3_C_2_ nanocomposites deposited on a cellulose (CS) paper. The Au/Ti_3_C_2_ nanocomposites uniformly wrapped around the cellulose fibers to provide a three-dimensional plasma SERS platform. The limit of detection (LOD) of CS/Au/Ti_3_C_2_ was as low as 10^−9^ M for 4-mercaptobenzoic acid(4-MBA) and crystal violet (CV), demonstrating good SERS sensitivity. CS/Au/Ti_3_C_2_ was used for in situ SERS detection of thiram on apple surfaces by simple swabbing, and a limit of detection of 0.05 ppm of thiram was achieved. The results showed that CS/Au/Ti_3_C_2_ is a flexible SERS substrate that can be used for the detection of thiram on apple surfaces. These results demonstrate that CS/Au/Ti_3_C_2_ can be used for the non-destructive, rapid and sensitive detection of pesticides on fruit surfaces.

## 1. Introduction

With the development of technology and highly integrated agriculture, more and more pesticides and fungicides are being used in agricultural production to control fungal infections and other diseases of crops [1,2]. However, the indiscriminate use of pesticides and fungicides has led to the contamination of soil and water and may endanger animal and human health through residues on crop surfaces. According to the U.S. Environmental Protection Agency (US-EPA) [3], more than 2.5 billion kilograms of pesticides is used globally each year, and the vast majority of those involved use pesticides to minimize the impact of diseases on crops [4]. Thiram is a dithiocarbamate fungicide that has been widely used to control fungal infections on a wide range of agricultural commodities, including seeds, fruits, vegetables and other crops. Because thiram acts on agricultural products by spraying, exposure to or consumption of thiram-contaminated crops, it has the potential to cause harm to a wide range of human and animal organs [5]. In past studies, a number of analytical methods have been used for the detection of thiram in order to reduce the risk of exposure to thiram. The more mature methods include high-performance liquid chromatography (HPLC), capillary electrophoresis (CE), mass spectrometry (MS) [1,6], etc. However, these detection methods usually have the drawbacks of being time-consuming, difficult-to-prepare samples, and requiring pre-treatment as well as specialized testing and sampling equipment [7]. Therefore, there is an urgent need for a convenient, fast and inexpensive sampling method for pesticide detection.

Surface-enhanced Raman scattering (SERS), as a hot technology in recent years, is expected to be used for rapid and convenient pesticide detection [8]. Raman scattering originates from the inelastic scattering of photons in molecules, in which energy is exchanged between the vibrational modes of the molecules and the photons [9]. Inelastically scattered photons with lower energy (Stokes Raman shift) and higher energy (anti-Stokes Raman shift) contain analyte-specific molecular information and have a wider range of applications. SERS detection is one of the most promising biosensing technologies, with high molecular specificity, ultra-high sensitivity and detection capabilities down to the single-molecule level. Gu, CY et al. [10] report the preparation of monolayer silica@gold core-shell nanoparticle (SiO_2_@Au NP) nanostructures, which had a limit of detection for CV of 10^−8^ M. Ma, LX et al. [11] successfully developed a label-free SERS sensor for simultaneous detection of acetamiprid and thiram using easily synthesized bimetallic core-shell Au@Ag nanoparticles. Pu, HB et al. [12] reported Au@Ag NRs with different shell thicknesses were prepared by coating different amounts of silver nitrate. Finally, the optimized Au@Ag NRs were used to detect thiram solution, and a low limit of detection of 0.003 mg/L was achieved. Also, the SERS substrate could be prepared by a physical method, S.V. Dubkov et al. [13] reported a Raman scattering enhancement in the red region of the spectrum when using an array of nanoparticles of the Ag–Cu eutectic system by physical sputtering.

In traditional SERS substrates, silicon, glass and metal-based materials account for a large proportion of rigid substrates due to their high sensitivity and stability [14]. In the sampling process of rigid substrates, it is usually necessary to drop the test material on the surface of the substrate or for the substrate to be submerged by the test material. For pesticide detection, especially for non-destructive SERS detection of pesticides on crop surfaces, the use of rigid substrates is limited. Therefore, there is a need for a new type of SERS substrate that can be used through a simple sampling method and an easy sample preparation process.

**Figure 1:**
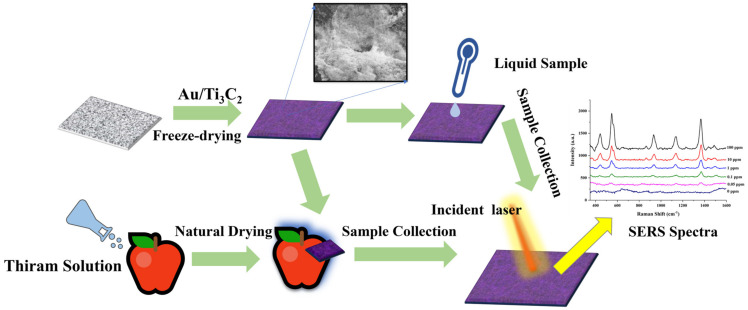
CS/Au/Ti_3_C_2_ for SERS detection of dye molecules and pesticides.

In our current work, we successfully synthesized Au/Ti_3_C_2_ nanocomposites by a self-assembly strategy and deposited them on cellulose paper by freeze-drying. Subsequently, CS/Au/Ti_3_C_2_ was used to detect CV and thiram, and thiram was detected on the surface of apples by simple wipe sampling (Figure 1).

## 2. Materials and Methods

### 2.1. Synthesis of CS/Au/Ti_3_C_2_

To synthesize Au/Ti_3_C_2_, a self-assembly method was employed. Firstly, Ti_3_C_2_ was synthesized following a procedure similar to that described in our previous report [15]. Gold nanoparticles (Au NPs) were synthesized by reducing HAuCl_4_ with sodium citrate, following a previously reported method [16]. Then, 200 μL of APTES solution was added dropwise to an ethanol solution of Ti_3_C_2_ and stirred for 24 h. Next, 335 μL of the APTES-Ti_3_C_2_ dispersion was mixed with 40 mL of Au NPs solution by stirring for 2 h at room temperature, followed by sonication and centrifuging. The Au/Ti_3_C_2_ solution was prepared by adding 4 mL of DI water to the residue.

Whatman 3 mm cellulose chromatography filter paper was cut into 5 mm × 5 mm size and fully soaked in Au/Ti_3_C_2_ solution. The soaked filter paper was pre-frozen at −20 °C and then freeze-dried in a vacuum freeze-dryer to remove water. Finally, CS/Au/Ti_3_C_2_ was obtained. The materials, reagents and detailed preparation methods are available in Supplementary Materials.

### 2.2. SERS Measurement

Raman spectra were acquired by a Raman spectrometer (LabRAM HR Evolution, HORIBA, Palaiseau, France) with a 50× lens.

For the detection of 4-MBA and CV, the CS/Au/Ti_3_C_2_ was fully immersed with an aqueous solution of the corresponding molecule, dried and measured. All assays were performed under a holographic grating at 1200 g/mm. The excitation laser was 633 nm at room temperature. The spot diameter was about 3 μm, and the laser power was about 0.2 mW. The accumulation times of the SERS spectra were 20 s and 25 s, respectively.

For the detection of thiram, a methanol aqueous solution with a volume ratio of 1:1 was used to dilute the thiram solution. The CS/Au/Ti_3_C_2_ was fully soaked with different concentrations of thiram solution and then dried and measured. The detection was carried out under a holographic grating at 600 g/mm. The excitation laser was 785 nm at room temperature. The spot diameter was about 4 μm, and the laser power was about 1.3 mW. The accumulation time of the SERS spectra was 10 s. The SERS spectra were measured with the help of the Raman spectrometer.

For the detection of thiram residues on apple skin, the apple skin was first cleaned using DI. Then, different concentrations of thiram solutions were sprayed evenly on the apple epidermis. We waited for the surface solution to dry completely and then prepared it for use. The CS/Au/Ti_3_C_2_ soaked in aqueous methanol solution was wiped well on the apple surface. The CS/Au/Ti_3_C_2_ was dried and used for SERS detection.

## 3. Results and Discussions

### 3.1. Characterization of CS/Au/Ti_3_C_2_

**Figure 2:**
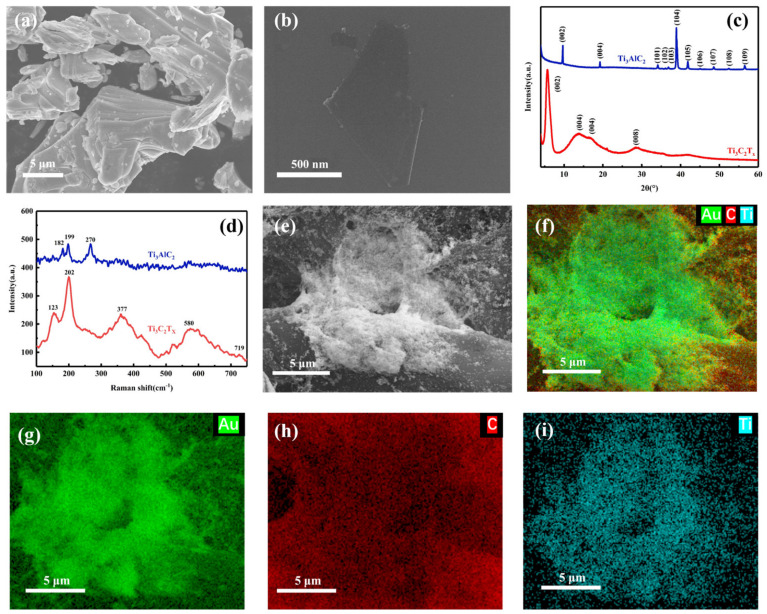
SEM of Ti_3_AlC_2_ (**a**) and monolayer Ti_3_C_2_T_x_ nanosheet (**b**). (**c**) XRD patterns of Ti_3_C_2_T_x_ and Ti_3_AlC_2_. (**d**) Raman signals of Ti_3_C_2_T_x_ and Ti_3_AlC_2_. (**e**) SEM of CS/Au/Ti_3_C_2_. Elemental mapping image of Au (**g**), C (**h**), Ti (**i**), and their overlay distribution (**f**).

As we previously reported, the Al atoms in the middle of Ti_3_AlC_2_ was etched by HF, which allowed Ti_3_C_2_T_x_ to be prepared. Ultrasonication further increased the layer spacing of Ti_3_C_2_T_x_, resulting in few layers of Ti_3_C_2_T_x_. SEM images of Ti_3_AlC_2_ (Figure 2b) show that Ti_3_AlC_2_ exhibits a stacked accordion-like structure, whereas the Ti_3_C_2_T_x_ nanosheets exhibited a large surface area and sharp edges after etching and presented a monolayer structure. Figure 2c demonstrates the XRD patterns of Ti_3_AlC_2_ and Ti_3_C_2_T_x_. The results show that the (104) plane peak at 2θ ≈ 39° disappears after the mixed etchant treatment, which proves that the Al atoms have been successfully etched [17]. In addition, the (002) diffraction peak of the Ti_3_C_2_T_x_ nanosheets broadens and shifts to a lower angle compared to the pristine Ti_3_AlC_2_. This confirms that the layer spacing of the Ti_3_C_2_ nanosheets has increased to form a monolayer nanosheet structure. The Raman spectra of Ti_3_AlC_2_ and Ti_3_C_2_T_x_ are shown in Figure 2d. The spectrum is divided into three regions: the flake region (about 150–250 cm^−1^), which corresponds to a group vibration of carbon, two titanium layers, and surface groups; the T_x_ region (about 210–500 cm^−1^), which represents vibrations of the surface groups; and the carbon region (about 530–780 cm^−1^). A_1g_ (Ti, C) at 182 cm^−1^ and 199 cm^−1^ vibrations of MAX phase shifted to 202 cm^−1^ because of etching and MXene formation. The disappearance of A_1g_ (Ti, Al) at 270 cm^−1^ is further evidence that the Al layer has been etched. The single-layer Ti_3_C_2_T_x_’s interlayer spacing becomes larger, which shifts the A_1g_ (C) peak to 719 cm^−1^ [18].

The Au NPs were synthesized by reducing HAuCl_4_ with sodium citrate. The Ti_3_C_2_ surface was modified with APTES and complexed with the Au NPs by amino dehydration condensation linkage. Finally, Au/Ti_3_C_2_ was deposited on cellulose fibers by freeze-drying. Figure 2e shows the SEM image of the CS/Au/Ti_3_C_2_ with Au/Ti_3_C_2_ uniformly wrapped around the fibers of the cellulose paper. In addition, an elemental map analysis (Figure 2f–i) showed that Ti, C and Au elements were uniformly distributed on the fibers of the cellulose paper. Therefore, these results indicate that Au/Ti_3_C_2_ was successfully adsorbed on the surface of the cellulose paper.

### 3.2. SERS Properties of CS/Au/Ti_3_C_2_

MXene and Au NPs have been reported to have excellent SERS performance [19,20,21]. Moreover, MXene has a large specific surface area and abundant surface functional groups, which allows the adsorption of target molecules. Charge transfer between molecules and MXene lead to enhanced Raman signals and enhanced localized surface plasmon resonance (LSPR) [15]. This makes it expected that it would be an excellent SERS substrate.

**Figure 3:**
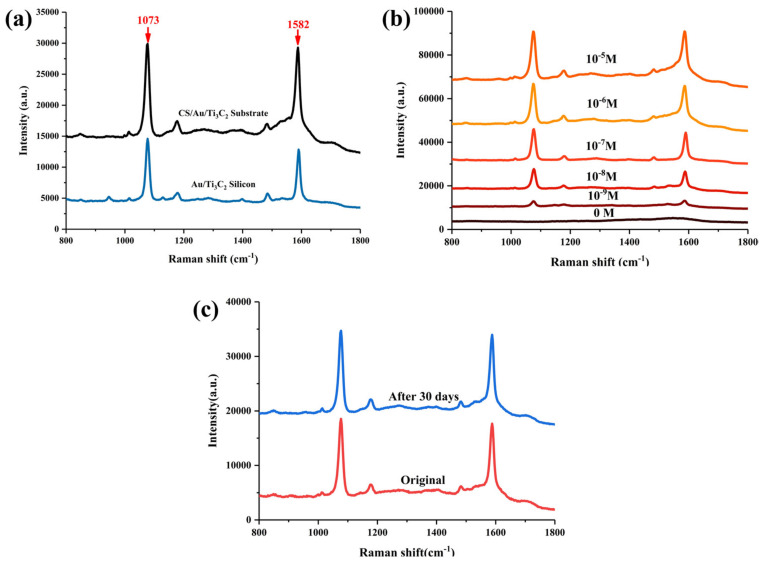
(**a**) SERS spectra of 10^−7^ M of 4-MBA on Au/Ti_3_C_2_ silicon and CS/Au/Ti_3_C_2_ substrates. (**b**) SERS spectra of different concentrations of 4-MBA measured by CS/Au/Ti_3_C_2_ substrate. (**c**) SERS spectra on CS/Au/Ti_3_C_2_ within 30 days.

4-MBA, a common Raman reporter molecule [22], was used to evaluate the SERS performance of CS/Au/Ti_3_C_2_. In order to more visually compare the difference between Au/Ti_3_C_2_ silicon and CS/Au/Ti_3_C_2_ substrates, both substrates were used to measure 4-MBA at 10^−7^ M. The experimental results are shown in Figure 3a. The SERS signal intensities of 4MBA molecular vibrational modes (C–S stretching mode: 1073 cm^−1^, benzene ring stretching mode: 1582 cm^−1^) [12] show that the CS/Au/Ti_3_C_2_ substrate has better signals of Raman marker bands than the Au/Ti_3_C_2_ silicon when detecting the same concentration of 4-MBA. In addition, Figure 3b shows the SERS spectra of 4-MBA in concentrations varying from 10^−5^ M to 10^−9^ M. According to previous research, the LOD of the assay is estimated based on the signal-to-noise ratio of detected spectral peaks greater than three. The signal-to-noise ratio is calculated according to the formula SNR= I_Ram_/√I_tot_, where I_Ram_ is the peak height of the analyte molecule at the lowest concentration and I_tot_ is the peak height of total Raman peak [23]. The signal-to-noise ratio for 4-MBA was calculated to be 38.41, which is much greater than 3. The results showed that the detection limit was as low as 10^−9^ M, which proved that CS/Au/Ti_3_C_2_ had good SERS performance. As a high-performing SERS substrate, an assessment of its stability is indispensable. 4-MBA at a concentration of 10^−7^ M was examined using freshly prepared CS/Au/Ti_3_C_2_ and CS/Au/Ti_3_C_2_ placed for 30 days, respectively (Figure 3c). The results showed that the marker bands detected in CS/Au/Ti_3_C_2_ after 30 days of placement did not show significant decline, which proved that the paper substrate has good stability.

### 3.3. SERS Detection of CV

Based on the previous study, CS/Au/Ti_3_C_2_ has been shown to have excellent SERS performance. In the practical application of SERS substrates, the detection of contaminants occupies an important position. CV, a triphenylmethane dye, is often used in the aquaculture industry due to its good bactericidal and parasite control properties [24]. However, the misuse of CV results in the possible presence of crystal violet residues in environmental water and aquatic products [25], which seriously endangers the environment and human health. Therefore, the SERS detection of on CV based on CS/Au/Ti_3_C_2_ was further developed.

**Figure 4:**
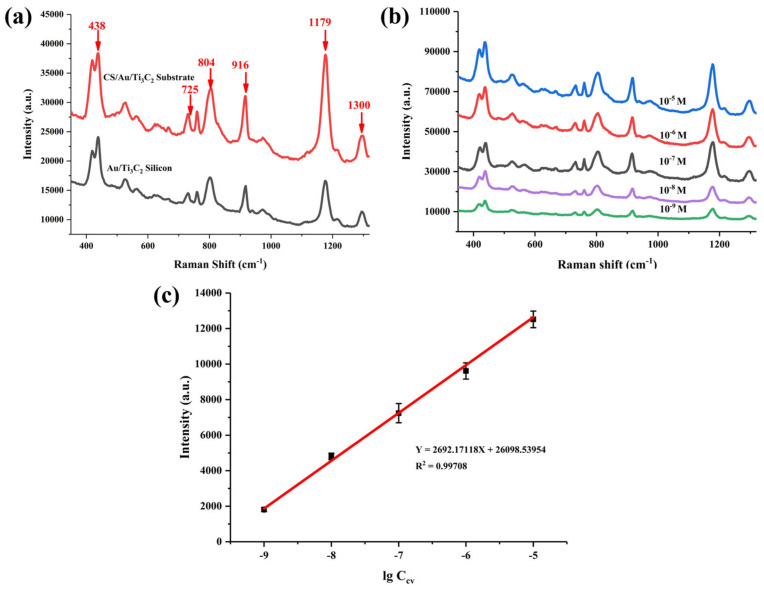
(**a**) SERS spectra of 10^−6^ M of CV on Au/Ti_3_C_2_ silicon and CS/Au/Ti_3_C_2_ substrates. (**b**) SERS spectra of different concentrations of CV measured by CS/Au/Ti_3_C_2_ substrate. (**c**) Plot of Raman mode intensity at 1179 cm^−1^ versus log values of concentration of CV (10^5^–10^9^ M).

Similarly, CV was used to assay the SERS performance of CS/Au/Ti_3_C_2_. A similar situation occurred with the detection of CV. As shown in Figure 4a, the CS/Au/Ti_3_C_2_ substrate has better SERS performance than the Au/Ti_3_C_2_ silicon for CV detection. Fingerprint bands in the spectra of CV—corresponding to C–C bending modes at 1300 cm^−1^; aromatic C–H bending modes at 1179, 916, 804, and 725 cm^−1^; and C–N-C bending modes at 438 cm^−1^ are observed, which agree well with the literature data [10,13,24]. As shown in Figure S1, the full spectrum up to 3000 cm^−1^ exhibits the full range of fingerprint bands of the CV.

Figure 4b shows the SERS spectra of CV in the concentrations range from 10^−5^ M to 10^−9^ M, and the mode intensity of the Raman marker bands was found to decrease with a decreasing concentration of CV. This implies that there may be a linear relationship between mode intensity and concentration. The calibration curve was established by monitoring the intensity of the marker band at 1179 cm^−1^ as a function of analyte concentration. The horizontal coordinates of the points on the calibration curve are lg C_CV_, and the vertical coordinates are the average of four consecutive measurements of the intensity of the marker band at that concentration. The fitting results are shown in Figure 4c, where a good linear correlation was established by plotting the logarithmic values of the marker band intensities and CV concentrations (R^2^= 0.99708). The detection limit of this method for CV molecules is as low as 10^−9^ M (SNR_CV_= 53.17). Therefore, the above experimental results demonstrate that the present CS/Au/Ti_3_C_2_ sensor exhibits excellent SERS performance for contaminant CV molecules.

Considering that the handheld Raman detectors on the market today basically use 785 nm as the excitation laser, the excitation laser of 785 nm was used in the detection of thiram in the actual samples. In addition, the 785 nm excitation laser was used for the detection of CV and 4-MBA. The experimental results show that both excitation lasers can realize the detection of CV and 4-MBA (Figures S2 and S3).

### 3.4. SERS Detection of Thiram

CS/Au/Ti_3_C_2_ has demonstrated the sensitivity of its SERS assay by immersion in the solution of the substance to be measured. However, a major advantage for flexible SERS substrates is that in situ detection can be achieved by simply wiping the surface of the object to be detected. This advantage of flexible substrates promises to make the detection of pesticide residues on crop surfaces faster and easier. It eliminates the need for specialized equipment and instruments for sample preparation.

**Figure 5:**
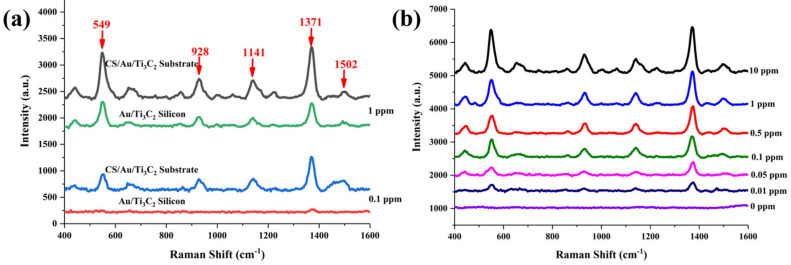
(**a**) SERS spectra of 1 ppm and 0.1 ppm of thiram on Au/Ti_3_C_2_ silicon and CS/Au/Ti_3_C_2_ substrates. (**b**) SERS spectra of different concentrations of thiram measured by CS/Au/Ti_3_C_2_ substrate.

To explore the capability of CS/Au/Ti_3_C_2_ in in situ Raman detection, a common spray-shaped pesticide [26], thiram, was used as a target molecule. Figure 5a shows the SERS spectra for the detection of thiram at 1 ppm and 0.1 ppm on Au/Ti_3_C_2_ silicon and CS/Au/Ti_3_C_2_ substrates, respectively. The mode at 549 cm^−1^ is assigned to the S-S stretching mode. The mode near 1371 cm^−1^ is attributed to CH_3_ deformation and CN stretching. The modes at 1141 cm^−1^ and 1502 cm^−1^ are assigned to CN stretching and CH_3_ wagging modes [27], respectively. When the SERS substrate is Au/Ti_3_C_2_ silicon, the detection of 1 ppm of thiram has a lower marker band intensity, and the detection of 0.1 ppm of thiram makes it almost impossible to observe the marker bands. In contrast, when CS/Au/Ti_3_C_2_ was used as the SERS substrate, both concentrations showed significantly stronger marker bands than those of the silicon-based one. This means that CS/Au/Ti_3_C_2_ still shows much higher detection ability than silicon substate in the detection of thiram. The detection limit of CS/Au/Ti_3_C_2_ was evaluated by measuring Raman spectra of different concentrations of thiram in methanol solution from 0 to 10 ppm. The results showed that the marker bands of thiram could still be detected even at concentrations as low as 0.01 ppm (SNR_thiram_ = 15.56) (Figure 5b).

**Figure 6:**
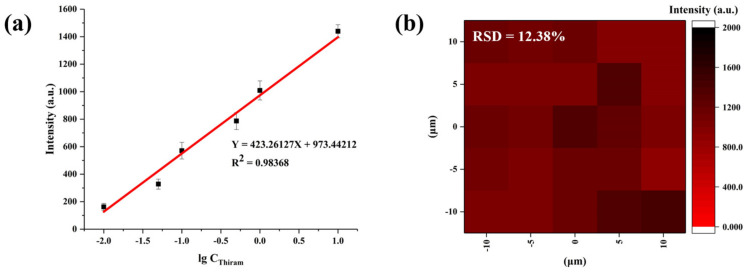
(**a**) The plot of Raman mode intensity at 1371 cm^−1^ versus the log values of concentration of thiram (0.01–10 ppm). (**b**) SERS intensity maps at 1371 cm^−1^ of 0.1 ppm of thiram.

The linear relationship between the concentration of thiram and the mode intensity of the characteristic peak and the spatial homogeneity were used to assess the SERS performance of CS/Au/Ti_3_C_2_ substrate. Similar to that in the detection of CV, a linear fit was performed using the lg C_thiram_ and the marker band intensity of thiram located at 1371 cm^−1^. As shown in Figure 6a, a good linear correlation (R^2^ = 0.98368) was established by plotting the log values of the SERS intensity and thiram concentration. In the evaluation of spatial uniformity, a 20 μm × 20 μm area on the CS/Au/Ti_3_C_2_ substrate was taken for SERS detection at 25 spots. The hotspot mapping is shown in Figure 6b, and the spatial homogeneity of the region was evaluated by relative standard deviation (RSD). The results showed that the RSD = 12.38% and the CS/Au/Ti_3_C_2_ had good spatial homogeneity.

**Figure 7:**
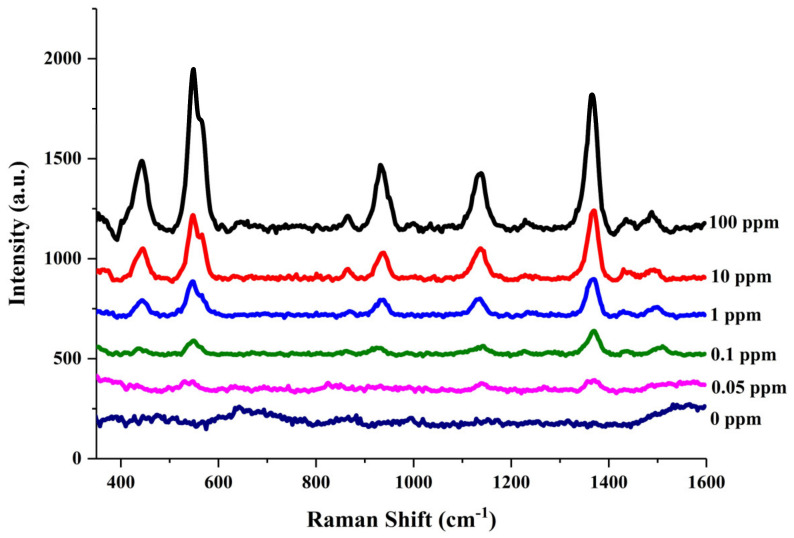
SERS spectra of different concentrations of thiram measured by CS/Au/Ti_3_C_2_ substrate with the simple wipe sampling method.

Further experiments were conducted to investigate whether the CS/Au/Ti_3_C_2_ flexible substrate prepared in this paper can be used for the detection of pesticide residues on crop surfaces by simple wiping. Briefly, the CS/Au/Ti_3_C_2_ substrate was moistened with a methanol solution and fully wiped on the surface of apples containing dried pesticide residues, waiting for the CS/Au/Ti_3_C_2_ to completely dry for SERS detection. The concentrations of thiram pre-sprayed on the apple surface were 100 ppm, 10 ppm, 1 ppm, 0.1 ppm and 0.05 ppm. The experimental results are shown in Figure 7, where CS/Au/Ti_3_C_2_ still reached the limit of detection, 0.05 ppm, with the simple wipe sampling method. The limit of detection was calculated to be 0.05 ppm, which is well below the maximum residue limit (MRL) in fruit of 7 ppm set by the U.S. Environmental Protection Agency (EPA). Therefore, the CS/Au/Ti_3_C_2_ flexible substrate is expected to be used for rapid and sensitive SERS detection of residue on the fruit surface.

## 4. Conclusions

In summary, facile synthesis of CS/Au/Ti_3_C_2_ was achieved and used as flexible SERS substrates. After freeze-drying, Au/Ti_3_C_2_ was uniformly and densely coated on the surface of cellulose fibers. Compared with Au/Ti_3_C_2_ silicon, CS/Au/Ti_3_C_2_ substrate has better SERS performance with a lower detection limit. The LOD of CS/Au/Ti_3_C_2_ substrate for 4-MBA and CV is as low as 10^−9^ M, showing good satisfactory sensitivity and chemical stability. Moreover, the flexible CS/Au/Ti_3_C_2_ substrate can be used for quantitative detection of thiram and achieved a good linear correlation (R^2^ = 0.98368) in the range of 0.01–10 ppm of thiram. The non-destructive Raman detection of thiram on the apple surface was realized by simple wiping of CS/Au/Ti_3_C_2_, and the LOD was 0.05 ppm. These results indicate that CS/Au/Ti_3_C_2_ has great potential as SERS substrate for the detection of pesticides in fruits.

## Data Availability

Data are contained within the article and Supplementary Materials.

## Supplementary Materials

The following supporting information can be downloaded at: https://www.mdpi.com/article/10.3390/nano14110987/s1.
