# Supplementary material for: Simple Synthesis of Cellulose-Based Nanocomposites as SERS Substrates for In Situ Detection of Thiram
Source: Nanomaterials (Basel). 2024 Jun 6;14(11):987. doi: 10.3390/nano14110987 (PMC11173843; doi:10.3390/nano14110987)
Supplement: Supplementary file 1 [file nanomaterials-14-00987-s001.zip › nanomaterials-3014973-supplementary.pdf]

Supplementary material

Simple Synthesis of Cellulose-Based Nanocomposites as SERS Substrates for In Situ Detection of Thiram

Boya Shi, Lian Kan, Yuliang Zhao, Shangzhong Jin*, Li Jiang*

College of Optical and Electronic Technology, China Jiliang University, Hangzhou 310018, P. R. China.

**Li Jiang; lijiang@cjlu.edu.cn*

**Shangzhong Jin; jinsz@cjlu.edu.cn*

Materials and reagents:

Lithium fluoride (LiF), (3-Aminopropyl) triethoxysilane (APTES, 98%), Hydrochloric acid (HCl, 35%-38%), ethanol (99.7%) and trisodium citrate ($C_6H_5Na_3O_7{\cdot}2H_2O$ 99.0%) were all purchased from Macklin (Shanghai, China). Titanium aluminum carbide powder (Ti_3AlC_2, 400 mesh) was purchased from FoShan XinXi Technology Co., Ltd. (Guangzhou, China). Chloroauric acid ($HAuCl_4$) was purchased from Adamas-beta Co., Ltd. (Shanghai, China). 4-mercaptobenzoic (4-MBA, 99.7%), crystal violet (CV, ≥90%)， methanol (≥99.8%) and thiram (analytical standard) were purchased from Sigma-Aldrich and stored at 4 ℃. Apples were purchased from local markets All reagents were analytical grade and were used without further purification.

Synthesis of Au/Ti_3C_2 solution

Briefly, 1.6 g of LiF was added to 20 mL of 9 M HCl, and then 1 g of Ti_3AlC_2 MAX powder was gradually added to the solution while stirring continuously at 35°C for 24 hours. The resulting mixture was then centrifuged at 3500 rpm for 10 minutes to remove the upper liquid layer. The remaining acidic suspension was washed by centrifugation (5 minutes at 3500 rpm) with deionized (DI) water until the supernatant reached a pH of approximately 6. The Ti_3C_2 precipitate was then dispersed in 100 mL of DI water and ultrasonicated for 30 minutes, followed by centrifugation for 60 minutes to obtain a dark green supernatant containing monolayer Ti_3C_2 suspension. This colloidal solution was stored at 4°C for future use.

1.5 mL of monolayer Ti_3C_2 suspension, 3.5 mL of DI water, and 5 mL of ethanol were mixed and stirred. Then, 200 μL of APTES solution was added dropwise to the mixture and stirred for 24 hours. After the reaction, the suspension was washed three times with DI water at 3500 rpm for 10 minutes to remove any impurities. The final suspension was then dispersed in DI water. Next, 335 μL of the APTES- Ti_3C_2 dispersion was mixed with 40 mL of Au NPs solution by stirring for 2 hours at room temperature, followed by sonication for 30 minutes. The mixture was then centrifuged at 3500 rpm for 20 minutes, and the supernatant was removed. The Au/Ti_3C_2 solution was prepared by adding 4 mL of DI water to the residue.

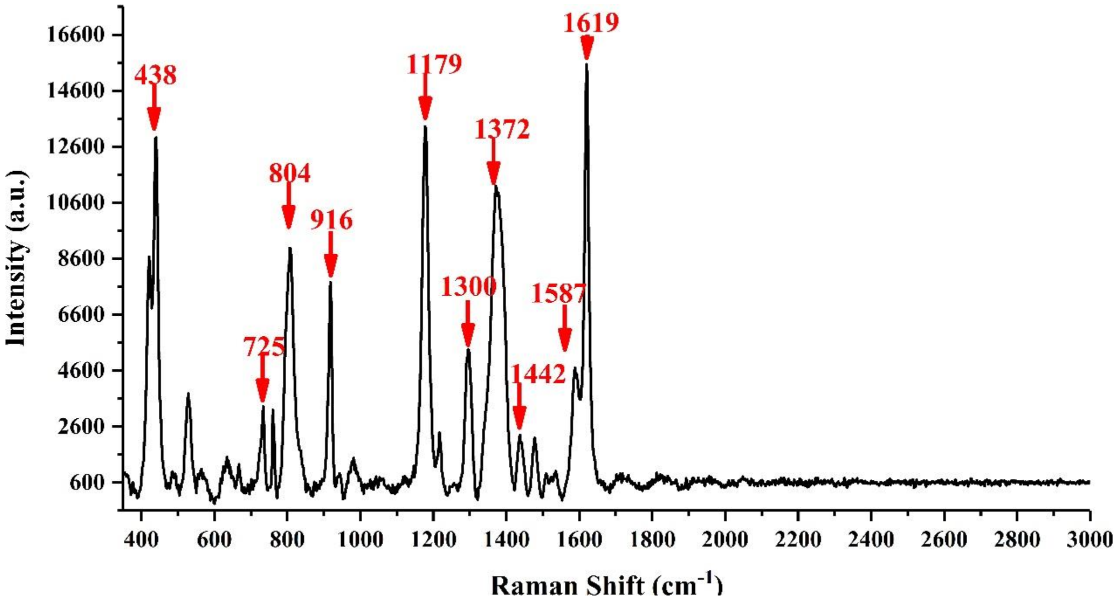

Figure. S1 SERS spectra of CV measured by CS/Au/Ti_3C_2 substrate.

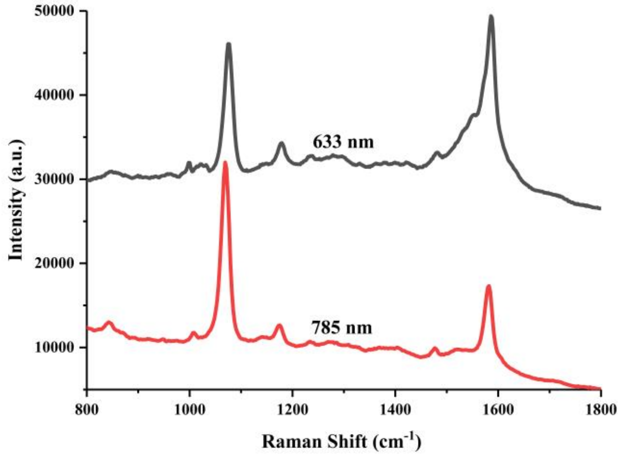

Figure. S2 SERS spectra of 4-MBA measured by excitation laser of 633 and 785 nm.

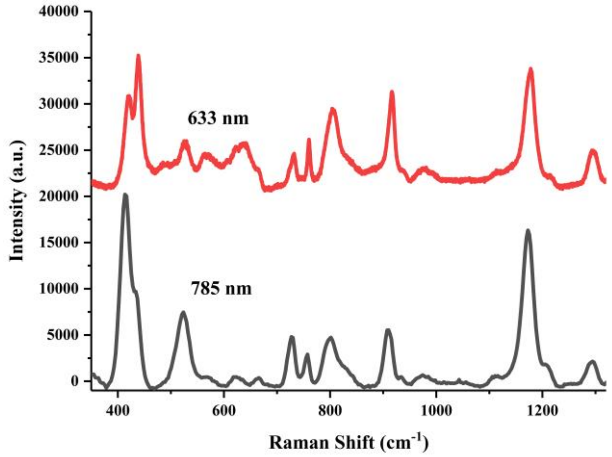

Figure. S3 SERS spectra of CV measured by excitation laser of 633 and 785 nm.
